# Functional changes in glutamate transporters and astrocyte biophysical properties in a rodent model of focal cortical dysplasia

**DOI:** 10.3389/fncel.2014.00425

**Published:** 2014-12-17

**Authors:** Susan L. Campbell, John J. Hablitz, Michelle L. Olsen

**Affiliations:** ^1^Department of Neurobiology, University of Alabama at BirminghamBirmingham, AL, USA; ^2^Department of Cell, Developmental and Integrative Biology, University of Alabama at BirminghamBirmingham, AL, USA

**Keywords:** GLT-1, astrocyte, electrophysiology, epilepsy, gliosis

## Abstract

Cortical dysplasia is associated with intractable epilepsy and developmental delay in young children. Recent work with the rat freeze-induced focal cortical dysplasia (FCD) model has demonstrated that hyperexcitability in the dysplastic cortex is due in part to higher levels of extracellular glutamate. Astrocyte glutamate transporters play a pivotal role in cortical maintaining extracellular glutamate concentrations. Here we examined the function of astrocytic glutamate transporters in a FCD model in rats. Neocortical freeze lesions were made in postnatal day (PN) 1 rat pups and whole cell electrophysiological recordings and biochemical studies were performed at PN 21–28. Synaptically evoked glutamate transporter currents in astrocytes showed a near 10-fold reduction in amplitude compared to sham operated controls. Astrocyte glutamate transporter currents from lesioned animals were also significantly reduced when challenged exogenously applied glutamate. Reduced astrocytic glutamate transport clearance contributed to increased NMDA receptor-mediated current decay kinetics in lesioned animals. The electrophysiological profile of astrocytes in the lesion group was also markedly changed compared to sham operated animals. Control astrocytes demonstrate large-amplitude linear leak currents in response to voltage-steps whereas astrocytes in lesioned animals demonstrated significantly smaller voltage-activated inward and outward currents. Significant decreases in astrocyte resting membrane potential and increases in input resistance were observed in lesioned animals. However, Western blotting, immunohistochemistry and quantitative PCR demonstrated no differences in the expression of the astrocytic glutamate transporter GLT-1 in lesioned animals relative to controls. These data suggest that, in the absence of changes in protein or mRNA expression levels, functional changes in astrocytic glutamate transporters contribute to neuronal hyperexcitability in the FCD model.

## Introduction

Development of seizures in children is often associated with focal cortical dysplasia (FCD; Krsek et al., 2009) with some manner of dysplasia present in up to 40% of intractable childhood epilepsies (Hagemann et al., 2006). These cortical malformations appear to arise from abnormal neuronal migration during brain development. This atypical migration leads to disruption of the cortical cytoarchitecture and formation of microgyria and heterotopias (D’Incerti, 2003). The mechanisms that contribute to epileptogenesis in patients with cortical malformations are not entirely clear. Improper maturation of neurons (Cepeda et al., 2006) and aberrant cortical synaptic connectivity that alter excitation-inhibition balance may in part underlie epileptiform activity (Zhou et al., 2009). In support of this, brain slices prepared from human dysplastic cortex display abnormal synaptic connections and increased excitability (Cepeda et al., 2006).

Many of the anatomical and electrophysiological characteristics of human FCD are reproduced following transcortical freeze-lesions in the newborn rat (Dvorák and Feit, 1977; Jacobs et al., 1996; Luhmann and Raabe, 1996; Hablitz and DeFazio, 1998). Using this well-characterized model, we and others have shown that adjacent to the lesion is a hyperexcitable zone which displays epileptiform discharges, alterations in neuronal NMDA receptors, and increases in tonic NMDA receptor-mediated currents (Jacobs et al., 1996; Campbell and Hablitz, 2005, 2008). These changes contribute to neuronal hyperexcitability in this model. Additional work has demonstrated that this hyperexcitability may be due in part to higher levels of extracellular glutamate resulting from changes in glutamate transporters (Campbell and Hablitz, 2008). The present work tests the hypothesis that increases in extracellular glutamate ([Glu^−^]_out_) arise in part from deficits in astrocytic glutamate uptake.

Astrocytes are charged with removing glutamate from the extracellular space (ECS) following neuronal activity and maintaining [Glu^−^]_out_ at low ambient concentrations. Normal brain function demands [Glu^−^]_out_ levels be kept low (1–2 µM) to avoid receptor desensitization or excitotoxicity (Cavelier et al., 2005). In the gray matter, this function is largely mediated by the astrocyte specific, Na^+^-dependent glutamate transporter EAAT2 (GLT-1 in rodents). Under physiological conditions, experimental evidence indicates it is difficult to overwhelm astrocytic glutamate transporters. High frequency stimulation for prolonged periods does not affect glutamate clearance from the ECS; Diamond and Jahr, 2000. Astrocytes in culture, which express reduced GLT-1 relative to astrocytes *in vivo*, can effectively reduce glutamate to ambient levels when challenged repeatedly with 100-fold glutamate increases over physiological [Glu^−^]_out_ levels (Ye et al., 1999). Despite this, elevated levels of extracellular glutamate have long been linked to seizures and epileptiform activity (Eid et al., 2008). These data suggest that for glutamate to rise significantly in the epileptic brain marked reductions in expression and or function of glial glutamate transporters must occur.

Changes in glutamate transporter expression have been observed in human temporal lobe epilepsy (Mathern et al., 1999; Crino et al., 2002), neocortical epilepsy (White et al., 2001) and tuberous sclerosis (White et al., 2001), as well as in experimental models of epilepsy (Miller et al., 1997; Nonaka et al., 1998; Mathern et al., 1999; Simantov et al., 1999; Crino et al., 2002). However, the functional changes that occur when glutamate transporter expression is altered have not been examined. Here we hypothesized that functioning of glutamate transporters in astrocytes would be decreased in the hyperexcitable zone adjacent to the freeze-induced microgyrus.

## Methods

### Lesion induction and slice preparation

All experimental protocols were in accordance with the NIH guidelines and were carried out with approval from the Animal Care and Use Committee of the University of Alabama at Birmingham. Every effort was made to minimize pain and discomfort. Focal freeze lesions were induced in postnatal day (PN) 1 Sprague-Dawley rat pups using methods previously described (Campbell and Hablitz, 2008). In brief, newborn rats were anesthetized by hypothermia (5 min on ice). A small incision in the skin was made to expose the skull. A cooled (~−60°C) copper rod (diameter: 1 mm) was placed on the surface of the skull near the midline for 3–5 s. After suturing the scalp, the animals were warmed and returned to their home cage. Sham-operated control rats received placement of a non-cooled rod. A recovery period of 21–28 days before further studies was used for both freeze-lesioned and sham-operated rats.

Slices were prepared as described previously (Campbell and Hablitz, 2008). Rats were anesthetized with isoflurane and decapitated. The brain was removed quickly and placed in ice-cold saline, containing (in mM): 125 NaCl, 3.5 KCl, 0.5 CaCl_2_, 3.5 MgCl_2_, 26 NaHCO_3_ and 10 D-glucose. Coronal neocortical brain slices (300 µm thick) were cut through the microgyrus and from corresponding areas of sham-operated and littermate controls. The slices were stored for 40–60 min at 37°C in saline similar to that described above but containing 2.5 mM CaCl_2_ and 1.3 mM MgCl_2_. They were then kept at room temperature until recording. Solutions were perfused into the recording chamber via an in-line heater which maintained temperatures between 30–32°C. Astrocytes were identified based on cell morphology, lack of spontaneous synaptic activity and absence of action potentials. In some instances, biocytin (Sigma-Aldrich, St. Louis, MO) was added to the pipette solution (0.5 mg/mL) for *post-hoc* identification and assessment of cell coupling.

### Whole-cell recording

Whole-cell, voltage-clamp recordings were made from astrocytes as described previously (Olsen et al., 2006) and pyramidal neurons (Campbell and Hablitz, 2008). For astrocytes the standard KCl pipette solution contained (in mM) 145 KCl, 1 MgCl_2_, 10 EGTA, 10 HEPES sodium salt, and pH was adjusted to 7.3 with Tris-base. CaCl_2_ (0.2 mM) was added to the pipette solution just before recording, resulting in a free calcium concentration of 1.9 nM. For neurons, the intracellular solution contained (in mM): 134 K-gluconate, 1 KCl, 10 HEPES, 2 Mg-ATP, 0.2 Na-GTP, and 0.5 EGTA. pH and osmolarity were adjusted to 7.4 and 285–290 mOsms, respectively. For all experiments described temperature was maintained between 30–32°C using an in-line heater. Astrocytes were held at −80 mV and neurons were held at −70 mV. Resting membrane potentials were measured ~1 min after whole cell access was obtained. Recordings were made from layer II/III pyramidal cells, chosen on the basis of their depth below the pial surface and location (0.3–1.5 mm lateral to the microsulcus). Series resistance and input resistance were carefully monitored during each experiment with a 10 mV hyperpolarizing voltage step. During astrocyte recordings whole-cell capacitance and series resistances were also measured directly from the amplifier, with the upper limit for series resistance being 12 MΩ and series resistance compensation adjusted to 80% to reduce voltage errors.

Evoked synaptic currents were recorded from pyramidal neurons and synaptic glutamate transporter currents (STCs) were recorded from astrocytes. Currents were evoked with a bipolar stimulating electrode (twisted pair of 25 µm Formvar insulated nichrome wires) positioned within 150–200 µm of the recording pipette. Current pulses 10–100 µA in amplitude and 50–100 µs in duration were used. A stimulation frequency of 0.05 Hz was used. Unless stated otherwise, all records of synaptic transporter currents shown represent the average of 10 consecutive responses. Control responses were recorded from astrocytes voltage clamped at −80 mV. TBOA (30 µM) and DHK (300 µM) were subsequently bath applied.

Exogenous glutamate was applied using a Picospritzer (Warner, Hamden, CT) as described previously (Gonzalez-Islas and Hablitz, 2003). Glutamate responses were recorded in astrocytes voltage-clamped at −80 mV in a saline containing 500 nM TTX, 100 µM CdCl_2_, 50 µM CNQX, 20 µM bicuculline and 50 µM AP5 to isolate glutamate transporter currents (Bergles and Jahr, 1997). A 500 msec puff of glutamate (500 µM) was pressure applied onto the voltage clamped astrocyte. The puffing solution contained 120 mM NaCl, 3.5 mM KCl, 1.3 mM MgCl_2_, 2.5 mM CaCl_2_, 25 mM HEPES, 10 mM glucose, 500 µM glutamate, 500 nM TTX, 100 µM CdCl_2_, 50 µM CNQX, 20 µM bicuculline and 50 µM AP5 (pH adjusted to 7.4). The puffer pipette was placed in the same focal plane as the voltage clamped cell and manipulated until a maximum response was elicited. Astrocytic responses were not observed in control experiments when glutamate was omitted from the pipette solution. All traces shown are the average of 3 or more consecutive applications of glutamate.

To isolate NMDA receptor-mediated EPSC’s (excitatory post-synaptic currents) bicuculline (10 µM) and CNQX (20 µM) were added to the ACSF bathing solution. Layer II/III pyramidal neurons were stepped from a potential of −70 mV to +50 mV and NMDA receptor- mediated currents were evoked with a bipolar stimulating electrode positioned within 150–200 µm of the recording pipette (Layer IV/V). Current pulses 10–100 µA in amplitude and 50 µs in duration were used. A stimulation frequency of 0.05 Hz was used. For these experiments a cesium based internal solution was used which contained in mM: 130 CsCl, 10 EGTA, 2 MgATP, 10 HEPES, 0.2 mm NaGTP, 5 mm QX314, pH adjusted to 7.3 with CsOH and an osmolarity of ~284 mOsms. TBOA (50 µM) was bath applied. All traces shown are the average of 3 or more traces.

### Drugs

D-(−)-2-amino-5-phosphonovaleric acid (D-APV), DL-threo-β-benzylozyaspartic acid (TBOA) tetrodotoxin (TTX), 6-Cyano-7-nitroquinoxaline-2,3-dione (CNQX), bicuculline (Bic), memantine and dihydrokainic acid (DHK) were purchased from Tocris Bioscience (Ellisville, MO, USA). These drugs were stored in frozen stock solution and dissolved in saline prior to each experiment. All other chemical and drugs were purchased from Sigma-Aldrich.

### Western blot analysis

Thick (0.7–1 mm) neocortical coronal brain slices containing the microgyrus and corresponding areas of sham-operated littermate controls were obtained. Coronal slices allowed for easy identification of the midline, microsulcus and the hyperexcitable zone. The corpus callosum also served as a landmark. Great care was taken to ensure punches were collected from equivalent regions in lesioned and sham operated animals. Tissue punches (1 mm diameter) were obtained from the microsulcus, the hyperexcitable region and the contralateral cortex and placed in ice cold homogenization buffer (100 mM Tris, pH 7.5, 1% SDS) supplemented with protease and phosphatase inhibitors (Sigma). The tissue was mechanically homogenized and then sonicated for 10 s. Tissue homogenates were centrifuged for 5 min at 12,000 g at 4°C. Protein quantification was performed on the supernatant using a DC protein assay kit from Bio-Rad (Hercules, CA). Protein was heated to 60°C for 15 min in an equal volume of 2X sample buffer (100 mM Tris, pH 6.8, 10% SDS in Laemmli-sodium dodecylsulfate, 600 mM β-mercaptoethanol). Equal amounts of protein were loaded into a 4–20% gradient pre-cast SDS gel (Bio-Rad). Gels were transferred using a constant 200 mA current for 2 h at room temperature. Membranes then were blocked in blocking buffer (10% dried milk in TBST). After three 10 min washes, membranes were developed with enhanced chemiluminesence (Amersham, Arlington Heights, IL) using an Image Station 4000 MM (Kodak). The blots were then stripped and re-probed with GLT-1 guinea pig (1:10,000), GFAP mouse (1:5,000; Millipore) and gapDH mouse (Abcam 1:1000) or actin rabbit (Sigma) antibodies for a loading control. Protein expression was quantitated using the Kodak Image Station normalizing total GLT-1 or GFAP protein to gapDH or actin expression in the same lane. Relative amounts of protein are reported.

### Quantitative PCR

Tissue punches were obtained as described above. The tissue was homogenized in a glass homogenizer with 800 µL of RNA STAT 60 (Friendswood, TX). Total RNA was isolated using the protocol described. The total RNA was diluted to a final concentration of 1 ug/uL and converted to cDNA using the VILO Superscript kit from Invitrogen. Quantitative real-time PCR using TaqMan specific probes for GLT-1, GFAP, CNX 43 and gapDH (Applied Biosystems) in 3 control and 6 injured animals were performed. All probes used throughout this study span exon boundaries and as such only amplify mRNA. Each sample was run in triplicate, and normalized to gapDH, and the comparative Ct method was used to calculate changes in gene expression.

### Immunohistochemistry

Animals were anesthetized with an intra-peritoneal injection of ketamine (100 mg/kg) and perfused with a 4% paraformaldehyde solution for 30 min. The brain was removed and stored in 4% paraformaldehyde. After washing in PBS, 50 µM neocortical sections containing the microgyrus and corresponding sections in sham operated animals were cut using a Vibratome (Oxford Instruments). Sections were blocked for 1 h in 10% horse serum and 0.3% Triton-X100 in PBS (PBS). Primary antibodies were diluted in blocking buffer (1:3 solution with PBS) and incubated with slices overnight. The sections were then washed three times in diluted PBS incubated with FITC or TRITC-conjugated secondary antibodies obtained from Molecular Probes for 60 min at room temperature. The slices were then washed two times with diluted PBS, incubated with DAPI (10–4 mg/ml; Sigma), and finally washed two times with PBS before being mounted onto glass coverslips. Fluorescent images were acquired with a Zeiss Axiovert 200 M. Confocal images were acquired on an Olympus Confocal FV 300 (Center Valley, PA). Distances from the pial surface and the midline to the hyperexcitable zone were used as landmarks for image acquisition. Similar measurements were used in control animals to ensure images were obtained from similar regions.

### Statistical analysis

Current responses to various voltage step and ramp protocols were analyzed and measured in Clampfit 8.0 (Molecular Devices). The resulting raw data were graphed and plotted in Origin 8.5 (MicroCal, Northampton, MA). When comparing 2 groups, two-tailed *t*-tests were performed as appropriate. All data are reported as average ± standard error of the mean with an asterisk (*) indicating that *p* ≤ 0.05. Statistical analysis was performed using GraphPad software (San Diego, CA), and *p*-values are reported in the text. Current amplitudes are reported as mean ± SE, with *n* indicating the number of cells sampled. For western blot quantification, the relative amount of Kir4.1 protein in each lane was normalized to its loading control and then normalized to the total amount of protein in control lanes. The data are reported as mean ± SE with *n* indicating the number of animals per condition.

## Results

### Neuronal hyperexcitability in the lesioned cortex is dependent on reduced astrocyte glutamate uptake

**Figure 1:**
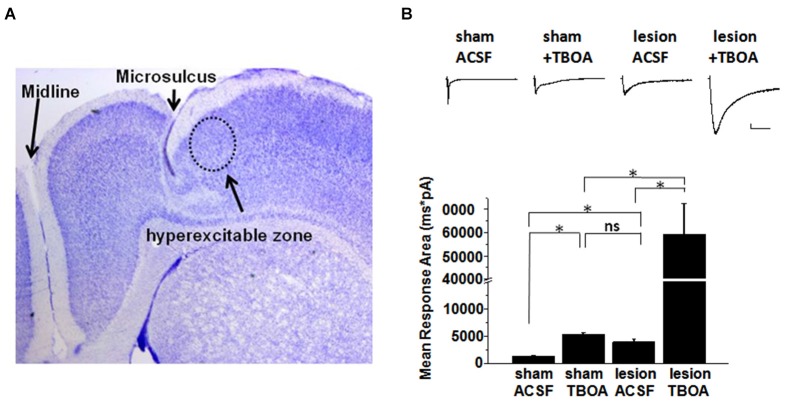
**Synaptic responses in pyramidal cells in slices from sham and lesioned animals. (A)** Representative image of a cresyl-violet stained slice from a lesioned animal showing the microsulcus. Dotted circle indicates the hyperexcitable zone. **(B)** Sample traces of evoked synaptic responses from a sham operated and lesioned animal. Responses obtained in (*top: left to right*) sham ACSF, sham after TBOA (30 µM) application, lesion neuron in ACSF and lesion neuron after TBOA (30 µM) application are shown. Bar graphs show quantification of the synaptic current response area following stimulation in sham and lesioned slices before and after TBOA application. Scale bar represents 200 pA and 500 mS.

**Figure 2:**
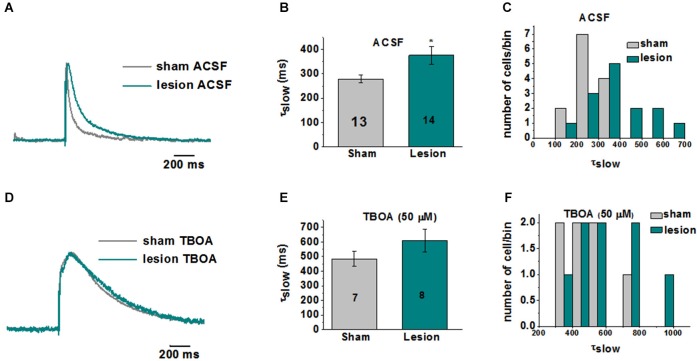
**Kinetics of NMDA receptor-mediated EPSCs are altered in layer II/III pyramidal neurons in lesioned cortex relative to sham-operated littermates. (A)** Representative traces (each recording is the average of 3 traces) indicate slowed NMDA receptor-mediated EPSC decay kinetics in lesioned animals. **(B)** Mean data from 13 sham and 14 lesioned neurons demonstrates a significant increase in *τ*_slow_ in neurons from the lesioned cortex relative to sham littermate controls. **(C)** Histograms of *τ*_slow_ indicate a greater number of cells in bins with larger *τ* values for the lesion group. **(D)** Representative traces showing no difference the NMDA response between the sham and lesioned neurons in the presence of TBOA (50 µM). **(E)** Mean data from 7 sham and 8 lesioned neurons indicate TBOA has a similar effect on the *τ*_slow_ NMDA receptor kinetics. **(F)** Histograms of *τ*_slow_ in TBOA indicate no difference between sham and lesion in *τ* bins.

A representative image of a cresyl violet stained cortical brain slice from a lesioned animal 24 days post lesion is shown in Figure 1A. The region adjacent to the microgyrus (a distance of 0.5 mm–2.5 mm, circled in black) has been termed hyperexcitable zone (Jacobs et al., 1996; Luhmann and Raabe, 1996). Neurons from this region display a lower threshold for evoking epileptiform activity and when stimulated have the capacity to generate abnormal electrical activity that spreads to surrounding tissue in the cortex (Jacobs et al., 1996; Luhmann and Raabe, 1996; Hablitz and DeFazio, 1998). The occurrence of these long lasting epileptiform discharges in the lesioned cortex are produced by a complex pattern of excitatory and inhibitory inputs (DeFazio and Hablitz, 2000). More recently, it has been demonstrated that the observed hyperexcitability is due in part to higher levels of extracellular glutamate which can be abrogated by blocking neuronal NMDA receptors (Campbell and Hablitz, 2008). Here we tested the notion that elevated glutamate is due in part to deficient astrocyte mediated glutamate clearance. In the first set of experiments we compared the currents elicited layer 2/3 pyramidal neurons in sham-treated slices when glutamate transporters were blocked with TBOA to those elicited in lesioned cortex neurons in normal ACSF. A brief electrical stimulation (50 us, 100 µA) applied in deeper cortical layers IV/V that was four times the stimulation required to elicit a threshold response was used. This was to ensure that only large current responses were induced, where glutamate transporter function would more likely be challenged (Campbell and Hablitz, 2004, 2008). In slices from sham treated animals, stimulation induced large responses in layer II/III pyramidal neurons with a mean response area of 1327 ± 125 pA*ms (*n* = 15 slices, Figure 1B). Bath application of TBOA (30 µM) significantly prolonged the response area (5081 ± 872 pA*ms, *n* = 15 slices, *p* < 0.001). Using the same stimulation protocol (four times the threshold) we elicited responses in the hyperexcitable zone of the lesioned cortex, which induced current responses with similar response area (3961 ± 872 pA*ms, *n* = 12 slices, *p* > 0.05) when compared to the response area in the sham slices in the presence of TBOA. When glutamate uptake was inhibited (TBOA 30 µM) in the lesioned cortex there was a pronounced increase in the response area (59214 ± 13271 pA*ms, *n* = 12 slices, *p* < 0.05) of the evoked epileptiform activity. This prominent increase in the response area in lesioned slices was independent of the initial stimulus intensity, since both low and high stimulus evoked events resulted in a marked prolongation of epileptiform activity (data not shown). To more directly assess the role of excess glutamate on neuronal excitability, we isolated NMDA receptor-mediate currents in control and lesioned animals in layer II/III pyramidal neurons (Figure 2). Here, neurons were stepped from a holding potential of −70 mV to +50 mV to remove the Mg^2+^ block. Stimulation in layer IV/V elicited outward NMDA receptor-mediated currents. Current decay for all 13 sham-operated neurons and 14 lesioned neurons were best fit by a sum of two exponentials. Representative, normalized traces demonstrate that the slow component of the decay (*τ*_slow_) was significantly increased in the lesioned animals relative to sham-operated littermates (Figure 2A). Here the *τ*_slow_ 279.3 +/− 16.3 in sham vs. 375.8 +/− 36.4 in lesioned animals (*n* = 13 sham-operated, *n* = 14 lesion, *p* = 0.0266, Figure 2B), suggesting prolonged glutamate in the ECS in the lesioned cortex following stimulation. Histograms of *τ*_slow_ indicate a greater number of cells in bins with larger *τ* values for the lesion group (Figure 2C). Although a trend was observed, there was no significant difference in the fast component of the NMDA decay constant (*τ*_fast_) between sham-operated and lesioned animals (66.7 +/− 10.3 (*n* = 13) and 96.9 +/− 14.3 (*n* = 14)). Application of TBOA to block astrocytic glutamate transporters significantly increased the *τ*_slow_ in both sham and lesioned animals (Figures 2B–D, 484.5 +/− 51.9 (*n* = 7) vs. 609.3 +/− 76.7 (*n* = 8), respectively) relative to ACSF. However, here was no statistical difference between the decay kinetics in sham and lesioned animals in the presence of TBOA (Figure 2B, *p* = 0.2142). There was also no difference observed in the fast component of the NMDA receptor-mediated current decay constant (*τ*_fast_) between sham-operated and lesioned animals in the presence of TBOA (123.2 +/− 27.2 and 139.0 +/− 29, respectively). Together, these results suggest that the increased excitability in the lesioned cortex is due, in part to elevated glutamate levels resulting from alteration in glutamate transporter function.

### Altered electrophysiological properties in hyperexcitable zone astrocytes

**Figure 3:**
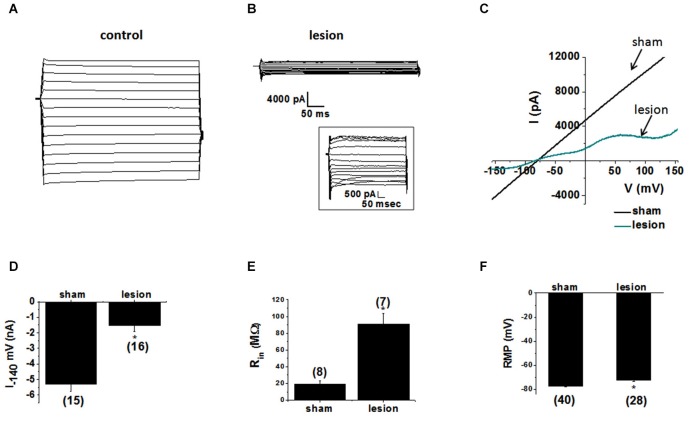
**Astrocytes in the hyperexcitable zone display altered electrophysiological properties. (A)** Representative whole-cell current responses to a voltage step protocol in an astrocyte in a slice from a sham-operated control and **(B)** a recording obtained from an astrocyte in slice from a lesioned animal. Inset shows current displayed at higher resolution. **(C)** Representative whole-cell currents from sham-operated (black) and lesioned (blue) astrocytes in response to a linear voltage ramp (−160 to +160 mV). **(D)** Bar graph showing differences in the mean current amplitude obtained from the voltage step protocol (at −140 mV) in control and lesioned astrocytes. **(E)** Bar graph showing the input resistance in astrocytes in lesioned animals was significantly higher (90.9 ± 13 MΩ) than that of control animals (19 ± 5 MΩ). **(F)** The resting membrane potential was significantly depolarized in astrocytes from lesioned animals (−71 ± 1.4 mV) relative to control astrocytes (−77 ± 0.77 mV).

To examine the contribution of astrocytic glutamate transporter dysfunction in the lesion cortex we first investigated the properties of the astrocytes in the hyperexcitable zone of lesioned cortex. Astrocytes recordings obtained from sham-treated animals, displayed large-amplitude, linear currents typical of passive astrocytes (Zhou et al., 2006), as shown in Figure 3A. Similar results were obtained in 15/15 astrocytes from sham-operated animals. In contrast, 13/16 astrocytes from the hyperexcitable zone of lesioned animals displayed prominent voltage-gated currents (Figure 3B). The inset shows currents at higher resolution. We also analyzed the response to voltage ramps. Large linear currents were elicited from control cells in response to a voltage ramp, as shown in the current-voltage plot in Figure 3C. Currents from astrocytes in the lesioned area were significantly smaller in amplitude and demonstrated pronounced voltage dependance. As shown in Figure 3D, peak inward currents at −140 mV in astrocytes from lesioned animals were significantly decreased compared to control (lesioned −1530 ± 373 pA, *n* = 16 vs. −5297 ± 471 pA, control, *n* = 15, *p* < 0.05). This decrease did not appear to be due to a change in cell coupling as no differences in the number of coupled cells between the two groups of astrocytes were observed (lesioned, *n* = 23.1± 2.8 vs. sham, *n* = 20.8 ± 3.4, *p* > 0.05). Supporting this, connexin 43, the primary connexin expressed in astrocytes, was not significantly reduced in lesioned cortex as assessed by qPCR (data not shown). This difference is attributable to a decrease in leak current in hyperexcitable zone astrocytes. Consistent with a reduced leak current, the input resistance in astrocytes from lesioned animals was significantly higher relative to astrocytes from sham-operated animals (90.9 ± 13 MΩ, lesioned, *n* = 7 vs. 19 ± 5 MΩ, controls, *n* = 8, *p* < 0.05, Figure 3E). The resting membrane potential (RMP) in astrocytes from lesioned slices also was significantly depolarized compared to those in controls (−71 ± 1.4 mV, lesioned, *n* = 40 vs. −77 ± 0.8 mV, control, *n* = 28, *p* < 0.05, Figure 3F).

### Reduced transporter currents in hyperexcitable zone astrocytes

**Figure 4:**
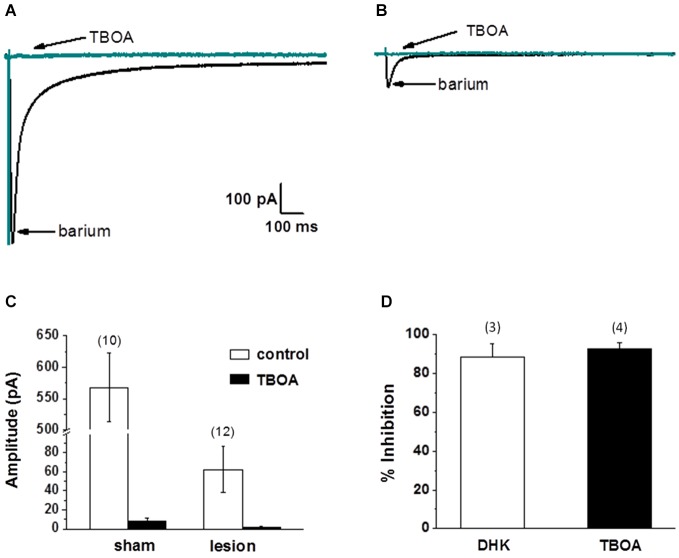
**Synaptically evoked glutamate transporter currents (STCs) are markedly reduced in astrocytes in lesioned animals. (A)** Specimen records showing evoked responses from a L2/3 astrocyte in a slice from a sham-operated animal following stimulation in cortical L4/5. Responses before and after bath application of TBOA are shown superimposed. TBOA effectively blocked the response. Each trace is average of ten consecutive responses. Experiments were performed in the presence of BaCl_2_ (100 µM) to inhibit a K^+^ uptake currents. **(B)** Similar to A, but from an astrocyte in a slice from a lesioned animal. Specimen records show that responses were smaller in amplitude relative to controls. Responses were TBOA sensitive. **(C)** Summary plots showing differences in STCs between astrocytes in slices from sham-operated and lesioned animals. STCs were significantly smaller in astrocytes from lesioned slices relative to astrocytes from sham-operated animals. **(D)** TBOA inhibited 92.8 +/− 3.3% (*n* = 4) of STC mediated currents while 88.6 +/− 6.7% (*n* = 3) of current was inhibited by DHK.

Thus far we have demonstrated that the neuronal response in lesioned animals in the hyperexcitable zone is similar to the response in sham treated animals in the presence of TBOA. We have also demonstrated that the NMDA receptor-mediated currents are significantly slower in lesioned animals, suggesting altered glutamate transport contributes to these differences. Furthermore, we have shown that astrocytes have markedly different electrophysiological properties in the hyperexcitable zone. In the next set of experiments, we examined astrocyte glutamate transporter currents in sham and lesioned animals. Release of glutamate from synaptic terminals can be detected in astrocytes by recording synaptically activated transporter currents (STCs; Mennerick and Zorumski, 1994; Bergles and Jahr, 1997; Diamond et al., 1998). STCs were evoked using a bipolar stimulating electrode located subjacent to the recorded astrocyte. Astrocytes in superficial cortical layers were voltage clamped at −80 mV and recordings were collected in saline containing 100 µM BaCl_2_ to block K_*IR*_ currents (Newman, 1993; Olsen et al., 2006). Sample recordings of currents evoked in an astrocyte from a sham-operated animal are shown in Figure 4A. In response to single shock stimulation, an inward current was observed. This response was completely blocked by TBOA (30 µM), identifying the current as a STC. When recordings were made in astrocytes located in the hyperexcitable zone (Figure 4B), responses were significantly smaller in amplitude and duration. STCs in these astrocytes were also blocked by TBOA. As shown in Figure 4C, STC amplitudes in lesioned animals were significantly smaller than those recorded in control astrocytes (568 ± 53 pA, *n* = 3 control animals, *n* = 10 slices with one cell per slice vs. 62 ± 24 pA, *n* = 4 lesioned animals, *n* = 12 slices with one cell per slice, *p* < 0.001). Two Na+ dependent glutamate transporters, GLT-1 and GLAST, have been identified in astrocytes with GLT-1 being the predominant transporter expressed in gray matter astrocytes (Rothstein et al., 1996; Tanaka et al., 1997; Regan et al., 2007), and accounting for over 90% of glutamate uptake (Tanaka et al., 1997). TBOA is non-specific, blocking both GLT-1 and GLAST in astrocytes. Therefore we repeated these experiments in sham-operated astrocytes using the GLT-1 specific inhibitor, DHK (300 µM). These data demonstrate that STC amplitudes were reduced by 92.8 +/− 3.3% (*n* = 4) by TBOA, whereas 88.6 +/− 6.7% (*n* = 3) of current was inhibited by DHK (Figure 4D). These data indicate that nearly all the synaptically evoked transporter current is mediated by GLT-1.

**Figure 5:**
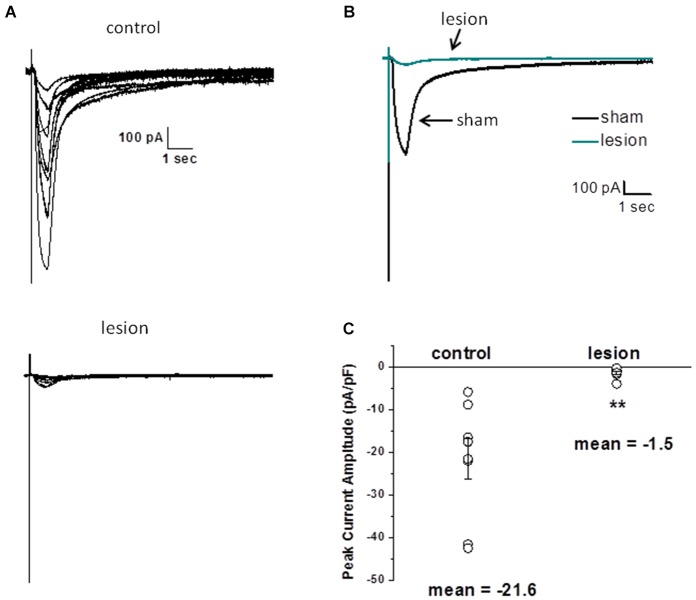
**Astrocyte responses to exogenously applied glutamate are reduced in lesioned cortex. (A)** Specimen records of glutamate evoked currents from astrocytes in slices from sham-operated (upper) or lesioned (lower) animals. Cells were voltage clamped at −80 mV and responses were evoked by a 500 µM, 0.5 s puff of glutamate. Bathing and puffing solutions contained 50 µM TTX, 200 µM AP5, 200 µM bicuculline, 250 µM CNQX and 100 µM CdCl. **(B)** Same data as in A but responses averaged across cells are shown. **(C)** Summary histogram showing that glutamate evoked responses, normalized for cell capacitance, were significantly reduced in the lesioned group.

Several factors, including alterations in local cortical connections in the dysplastic cortex, differences in threshold for synaptic activation and/or release properties, and changes in the astrocyte morphology could contribute to the differences in STC amplitudes described above. Therefore, we next examined astrocyte responses to exogenously applied glutamate. For these experiments, recordings were obtained in the presence of 500 nM TTX, 100 µM CdCl_2_, 50 µM CNQX, 20 µM bicuculline and 50 µM AP5 to minimize neuronal activity, release of endogenous glutamate and isolate glutamate transporter currents (Bergles and Jahr, 1997; Grass et al., 2004). Glutamate (500 µM, 500 msec) was pressure applied to astrocytes voltage clamped at −80 mV. The puffer pipette was visually placed in the same focal plane as the voltage clamped cell at a distance that elicited a maximum response. Specimen records from 8 control astrocytes (upper) and 7 astrocytes from the lesioned cortex (lower) are shown superimposed in Figure 5A. The average of these responses across cells is shown in Figure 5B. There was no statistical difference in cell size between the two groups, as measured by cell capacitance (control 21 ± 3 pF and lesion 23 ± 4pF, *p* > 0.05). The changes in STC current and glutamate uptake currents were nearly 10-fold smaller in lesion animals relative to sham operated animals. Normalizing glutamate transporter current to whole cell capacitance, which measures the amount of current flow per unit area of membrane, we observed transporter currents were significantly less in the hyperexcitable zone relative for that observed in sham operated animals (1.5 ± 0.46 pA/pF, *n* = 7, lesioned vs. 21.6 ± 4.8 pA/pF, *n* = 8, control, *p* < 0.05, Figure 5C). These data suggest that glutamate accumulation during epileptiform discharges in lesioned cortex would be less effectively cleared by astrocytes.

### Glutamate transporter protein and mRNA expression is unchanged in the hyperexcitable zone

The observed decreases in STCs and responses to exogenously applied glutamate suggest a functional decrease in the ability of transporters to take up extracellular glutamate. Although functional glutamate transporter currents have not been directly examined in models of FCD, previous work has examined glutamate transporter expression with variable results (Proper et al., 2002; Ulu et al., 2010). We therefore examined GLT-1 protein and mRNA expression.

**Figure 6:**
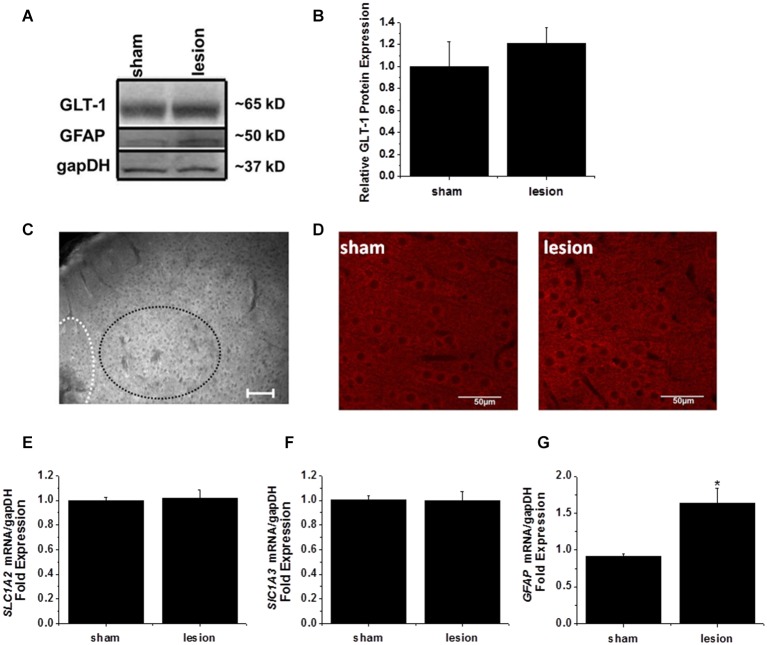
**GLT-1 protein and mRNA expression levels were unchanged in the lesioned animals. (A)** A representative Western blot demonstrating no change in GLT-1 expression in the hyperexcitable zone of lesioned tissue relative to tissue obtained from the same region in sham-operated animals. These blots were also stripped and re-probed with gapDH as a loading control and the astrocyte intermediate filament GFAP. **(B)** Plot of relative GLT-1 expression in lesioned tissue relative to control tissue. GLT-1 expression is normalized to the loading control gapDH. No significant difference was observed. **(C)** Wide field fluorescent microscopy was used to image the lesion, hyperexcitable zone and surrounding areas. No gross changes in GLT-1 expression were observed (scale bar = 100 µm). **(D)** Confocal imaging from a *z*-stack (60 sections, 1 µm/section) from the hyperexcitable region indicating GLT-1 immunoreactivity in a sham operated and lesioned animal (scale bar = 50 µm). **(E)** mRNA expression of GLT-1 (*SLC1A2*) was also unchanged in the hyperexcitable zone from lesioned animals when compared to tissue obtained from sham-operated animals (each sample ran in triplicate). **(F)** GLAST mRNA levels were not significantly different between groups. **(G)** GFAP expression was significantly elevated in the hyperexcitable zone (1.6 fold) relative to control tissue obtained from the same region.

A 1 mm diameter tissue punch was used to obtain cortical samples 0.7–1 mm thick from the hyperexcitable zone in lesioned animals and comparable areas in sham operated controls. A representative western blot is shown in Figure 6A. GLT-1 immunoreactivity was unchanged in the hyperexcitable zone relative to tissue from sham-operated controls. Quantification from four sets of animals is shown in Figure 6B. These results demonstrate there is no significant difference in GLT-1 protein expression between the hyperexcitable zone of lesioned animals and sham tissue when normalized to a loading control.

The lack of any appreciable decrease in GLT-1 protein expression prompted us to explore the possibility that a mislocalization of GLT-1 protein was responsible for the profound reduction in astrocyte glutamate uptake currents. As a first step, we performed wide-field fluorescent microscopy to examine GLT-1 expression in lesion and sham animals. We were unable to detect discernible changes in GLT-1 expression at the hyperexcitable zone relative to surrounding regions by wide field fluorescent microscopy (Figure 6C). No significant differences were observed using high powered confocal microscopy comparing GLT-1 expression patterns in hyperexcitable zone from lesion and sham operated animals (Figure 6D).

In a separate cohort of animals, tissue punches were collected from the same region for mRNA extraction and quantitative PCR studies. qPCR results indicated there was no significant difference in GLT-1 (SLC1A2) transcripts between sham operated and lesioned animals (Figure 6E). We also observed no significant difference in GLAST (SLC1A3) mRNA expression in the hyperexcitable zone relative to that observed in sham-operated animals (Figure 6F). GFAP mRNA expression, however, was significantly elevated in the lesioned animals hyperexcitable zone (1.5 fold relative to sham operated animals, Figure 6G). Expression of GFAP mRNA was elevated to a much higher extent at the lesion itself (7-fold increase over control, data not shown). While not exhaustive, these data, together with protein expression and immunohistochemistry data suggest there are no gross differences in GLT-1 expression levels.

## Discussion

In the present study, we demonstrate that astrocytes in slices from dysplastic neocortex display decreased glutamate transporter currents in response to both synaptic stimulation and direct application of glutamate. Additionally, astrocytes in the hyperexcitable zone display altered biophysical properties, including significantly depolarized RMPs, higher input resistances and reduced leak currents. Biochemical studies, however, did not indicate differences in GLT-1 expression. These data provide evidence for functional changes in glutamate transporter function in dysplastic cortex. These alterations could contribute to elevated extracellular glutamate levels and hyperexcitability in cortical dysplasia.

### Changes in astrocyte biophysical properties

Distinct patterns of currents are found in hippocampal (D’Ambrosio et al., 1998; Zhou and Kimelberg, 2000; Zhou et al., 2006) and neocortical astrocytes (Bordey et al., 2001). One population of glial cells demonstrates nearly linear current-voltage (I-V) relationships whereas another group displays outwardly rectifying I-V curves. Astrocytes with voltage activated inward and outward currents are observed more frequently earlier in postnatal development (Zhou et al., 2006). The presence of a linear I-V curve appears to be an intrinsic astrocyte property and not dependent of gap junction development (Schools et al., 2006). In the present study, linear currents typical of passive astrocytes were seen in recordings from control animals whereas voltage-dependent currents were prominent in the lesioned group (>80%). This was not due to differences in coupling as measured by dye coupling or connexin expression in the lesioned group. The observed decrease in leak currents and associated increase in input resistance may have allowed detection of previously obscured voltage-dependent currents in the dysplastic cortex. Numerous studies have attempted to identify the “leak” or “passive” current that dominates the K^+^ conductance in mature astrocytes. The primary candidates are the weakly rectifying (TWIK)-related acid sensitive K^+^ channel, TASK-1, the two-pore domain K^+^ channels (K2P) TWIK-1 and TREK-1 and the weakly rectifying potassium channel Kir4.1. Using a bacterial artificial chromosome (BAC) transgenic mouse line where EGFP expression is directly related to Kir4.1 promoter activity, Tang et al. demonstrated a relatively large contribution (>60%) of the overall passive current in attributable to Kir4.1 (Tang et al., 2009). Interestingly this was observed only when gap junction communication was inhibited. Heterologously expressed TWIK-1 and TREK-1 channels, share many of the pharmacological characteristics of the passive conductance observed in hippocampal astrocytes including inhibition with quinine, moderate inhibition by low extracellular pH, and a low sensitivity to Ba^2+^ (Zhou et al., 2009). It was proposed that a disulfide linked heterodimer of TWIK-1 and TREK-1 may be responsible for the large leak conductance in astrocytes (Hwang et al., 2014). However, a recent study using TWIK-1 knockout mice indicated that TWIK-1 contributes little to the whole cell current as it is largely retained in intracellular compartments or organelles. Only ~5% of the total TWIK protein is trafficked to the plasma membrane (Wang et al., 2013). Finally, TASK channels were recently ruled out as a molecular candidate due to differences in pharmacology between astrocyte currents in hippocampal slices and that which has been previously reported for TASK channels (Chu et al., 2010). Given the overlapping pharmacology and similar biophysical characteristics of leak or weakly rectifying potassium channels that mediates these large amplitude currents in astrocytes we did not pursue the molecular identification of the channel. Although K^+^ currents were not the focus of this study, we believe the loss of the leak current warrants future investigation as the reduced inward K^+^ current in astrocytes from lesioned slices likely has a significant effect on the ability of astrocytes to take up potassium ion from the ECS contributing to overall neuronal excitability. Previous studies have indicated that astrocytes respond to neuronal activity or stimulation with large amplitude inward currents that can be dissected into two components with different kinetics; a fast glutamate uptake component and a relatively slow potassium uptake component (Olsen, 2012; Sibille et al., 2014). The potassium uptake currents comprise the majority of the astrocytic response and are mediated by barium-sensitive Kir4.1 mediated current (Sibille et al., 2014). We have previously shown that the amplitude of the inward K^+^ current is directly proportional to efficiency of K^+^ uptake by these cells (Olsen et al., 2007). It can be surmised that the loss of the leak conductance from astrocytes in the lesioned animals would contribute to increased overall excitability, especially when coupled with elevated or dysregulated extracellular glutamate.

### Clearance of glutamate from the extracellular space

The clearance of glutamate from the ECS following neuronal activity and the resultant curtailment of glutamatergic transmission occurs primarily via the Na+-dependent glutamate transporters GLAST/EAAT1 and GLT-1/EAAT2. GLT-1 and GLAST are preferentially but differentially expressed in astrocyte populations throughout the CNS. In adult cortical gray matter, GLT-1 mediates the majority of glutamate clearance (Rothstein et al., 1996; Tanaka et al., 1997; Regan et al., 2007). Protein expression and mRNA transcripts for GLT-1 in astrocytes increase robustly from birth to young adulthood (P0–P30). In rodents, the most significant increases in GLT-1 expression occur during the first two postnatal weeks (Furuta et al., 1997; Regan et al., 2007). Correspondingly in humans, the most significant increase in EAAT2 occurs postnatally before 2 years of age (Lauriat et al., 2007b).

Glutamate uptake is achieved against a ~1000-fold concentration gradient between [Glu^−^]_out_ and [Glu^−^]_in_. This is achieved by coupling glutamate transport to Na^+^ and K^+^ transport down their respective concentration gradients and the hyperpolarized astrocyte membrane potential (Anderson and Swanson, 2000). Given that in our experiments the astrocyte membrane was voltage-clamped at −80 mV, the changes in membrane potential observed in astrocytes from the dysplastic cortex cannot account for the observed reduction in glutamate transporter currents. However, the depolarized membrane potential of astrocytes in lesioned animals could contribute to reduced glutamate uptake.

Our data clearly indicate significant alterations in glutamate transporter function. We observed a near 10-fold reduction in TBOA sensitive transporter function. Furthermore, application of DHK, a GLT-1 specific non transportable inhibitor, suggests ~90% of the currents in both sham-operated and lesioned astrocytes are mediated by GLT-1. Application of the same concentration of glutamate in puffing experiments ruled out the possibility that the reduced glutamate transporter responses in the lesioned astrocytes were the result of less glutamate release following stimulation in lesioned animals. Inefficient clearance of glutamate by astrocytic glutamate transports following electrical stimulation contributed to an enhanced hyperexcitability in the dysplastic cortex, which can be explained in part by altered kinetics of the NDMA receptor-mediated currents.

We postulated that the loss in GLT-1 transporter function would be paralleled by decreased protein and mRNA expression. To our surprise, this is not what we observed. Given our data, it seems likely that a change in transporter effectiveness may underlie the observed decreases in evoked transporter currents. The activity of glutamate transporters is affected by a variety of neuromodulators and second messenger systems. Decreases in transporter activity are induced by caffeine (Shin et al., 2013) and neurosteroids (Kang et al., 2010; Na et al., 2012) whereas pregabalin increases responsiveness (Ryu et al., 2012). Glutamate uptake in astrocytes has been shown to be potentiated by selective pharmacological stimulation of a transgenic Gq G-protein coupled receptor targeted specifically to astrocytes and activation of mGluRs on astrocytes enhances STCs (Devaraju et al., 2013). It should be noted that several splicing isoforms of GLT-1 have been identified, including multiple 5′ splice variants (Rozyczka and Engele, 2005) and isoforms in which entire exons are deleted (Lauriat et al., 2007a). The antibodies and qPCR probes used in this study would not have delineated between different spice variants which may exhibit functional differences in uptake. Furthermore, alterations in signaling molecules such as PKC, which alter GLT-1 trafficking to the plasma membrane (Kalandadze et al., 2002) may occur in the hyperexcitable zone. While our immunohistochemical studies indicate no gross changes in GLT-1 transporter immunoreactivity, they do not address transporter localization, or significant changes in astrocyte morphology that may affect glutamate import. Another possibility is that the conditions in the dysplastic cortex are favorable for glutamate transporters to function in reverse. Traditionally, application of extracellular K^+^ has been used to study reverse glutamate transport (Szatkowski et al., 1990) In the lesioned cortex, during epileptiform discharges there is elevated extracellular glutamate levels leading to elevated extracellular K^+^, coupled with our reported depolarized membrane potential in glia cells would create the perfect environment for transporters to function in reverse (Brew and Attwell, 1987). Indeed studies have shown that reverse glutamate transport occurs under some pathophysiological conditions including brain ischemia (Rossi et al., 2000). Our data shows that blocking glutamate transporters in the lesioned cortex exacerbated evoked epileptiform discharges, suggesting the remaining glutamate transporter function present on astrocytes in the freeze lesion plays a significant role in glutamate handling. Because glutamate transporters are only reduced in the lesion cortex and not completely abolished, small changes in residual transporters may play a significant role in glutamate accumulation in response to stimulation leading to a larger neuronal response. These small changes in residual transporters could result in larger changes in network activation. Inhibition of glutamate transporters may lead to further elevation in extracellular glutamate levels in the more susceptible areas surrounding the lesion leading to over-activation glutamate receptors and alteration of other glutamate-mediated mechanisms. Furthermore, glutamate independent mechanism not directly addressed here may also play a role in the increased excitability including; (1) changes in glial and neuronal morphology which modify the ECS and diffusion of glutamate and other excitatory molecules; (2) neuronal TBOA-sensitive transporters which may play a more significant role in the lesioned cortex; and (3) elevated extracellular K^+^ which plays a significant role in neuronal excitability.

### Glutamate transporters and cortical dysplasia

Elevated levels of extracellular glutamate have been reported in patients with epilepsy (During and Spencer, 1993; Cavus et al., 2005). The results of studies examining glutamate transporter expression in epilepsy are diverse. Increases and decreases in transporter expression have been reported both in resections from patients with epilepsy and in animal models (Mathern et al., 1999; Meldrum et al., 1999; Crino et al., 2002; Proper et al., 2002). Investigations of human temporal lobe epilepsy, tuberous sclerosis and neocortical epilepsy reported an increase in EAAC1 expression (Mathern et al., 1999; White et al., 2001; Crino et al., 2002). In patients with cortical dysplasia, glutamate transporters were differentially expressed in dysplastic neurons compared to balloon cells (González-Martinez et al., 2011). Expression of EAAC1 is decreased in the *in utero* MAM cortical dysplasia model (Harrington et al., 2007). A consistent pattern of change in glutamate transporters in epilepsy has not been apparent in these studies using immunocytochemistry and Western blotting. However, it is important to note that these studies do not measure functional transporter activity. Decreases in the ability to remove exogenously applied glutamate, measured by glutamate biosensor imaging, have been reported in freeze lesioned cortex (Dulla et al., 2012). This result is consistent with our finding of reduced transporter currents in astrocytes and suggests that functional alterations in glial glutamate transporters occur in freeze lesioned neocortex.

## Author contributions

Susan L. Campbell performed whole cell voltage clamp electrophysiology in neurons and astrocytes, immunohistochemistry and contributed to the writing of the manuscript. John J. Hablitz oversaw aspects of experimental design, implementation and writing of the manuscript. Michelle L. Olsen performed whole cell voltage clamp electrophysiology in astrocytes, neurons, Western blots, qPCR, immunohistochemistry and writing the manuscript.

## Conflict of interest statement

The authors declare that the research was conducted in the absence of any commercial or financial relationships that could be construed as a potential conflict of interest.
